# Interface-engineered Caco-2 cell culture on a collagen-coated liquid–liquid interface in a microfluidic device

**DOI:** 10.3762/bjnano.17.53

**Published:** 2026-06-11

**Authors:** Satoru Kuriu, Soo Hyeon Kim

**Affiliations:** 1 Institute of Industrial Science, The University of Tokyo, 4-6-1 Komaba, Meguro-ku, Tokyo 153-8505 JAPAN

**Keywords:** Caco-2, collagen, FC-43, gut-on-a-chip, liquid–liquid interface

## Abstract

Epithelial tissues form selective barriers essential for physiological homeostasis. Conventional in vitro models rely on solid substrates, which limit the physicochemical flexibility of the cellular microenvironment. Here, we introduce a microfluidic platform in which a collagen-coated liquid–liquid interface formed between perfluorocarbon (FC-43) and culture medium serves as a substrate for epithelial cell adhesion. By culturing Caco-2 cells in the device, we show that the liquid interface supports cell attachment and the formation of monolayers. Immunofluorescence observation reveals the development of tight junctions and organized actin cytoskeletons, indicating early-stage epithelial maturation. Our new microfluidic system enables the formation of stable liquid–liquid interfaces that serve as viable and flexible substrates for epithelial cell culture, offering new opportunities for multiphase microfluidic models of epithelial barriers.

## Introduction

Epithelial tissues form selective barriers that regulate the transport of molecules, ions, and gases; they play an important role in physiological homeostasis [1]. In vitro models of epithelial barriers, particularly culturing Caco-2 cells, have been widely used to study the function of the epithelial tissues [2–4]. The microfluidic culture system known as gut-on-a-chip enables the cultivation of Caco-2 cells under conditions that more closely recapitulate the in vivo intestinal environment. By precisely controlling fluid flow and inducing mechanical stimuli, this platform provides physiologically relevant mechanical cues that are difficult to reproduce in conventional static culture systems [5–7]. Gut-on-a-chip platforms recapitulate the apical-basolateral compartmentalization of the human intestinal epithelium by dividing upper and lower microchannels with a solid porous membrane, upon which intestinal epithelial cells are cultured to establish a polarized epithelial barrier. Although porous membranes enable compartmentalization, they may also impose non-physiological diffusion barriers and mechanical constraints.

Liquid–liquid interfaces provide a mechanically soft and dynamically reconfigurable environment that more closely mimics native cellular microenvironments than rigid solid substrates. Unlike conventional solid culture surfaces, liquid–liquid interfaces offer a distinct platform for cell culture, enabling a scaffold-free and mechanically tunable environment that eliminates the rigidity and pore-related constraints inherent to synthetic solid membranes [8–10]. Importantly, culturing cells at a liquid–liquid interface provides a unique opportunity to decouple biochemical signaling from substrate-imposed mechanical constraints, thereby enabling more physiologically relevant investigation of epithelial barrier formation, transport functions, and mechanobiological responses that are otherwise difficult to isolate in conventional solid-supported systems. Moreover, when functionalized with extracellular matrix proteins, such interfaces can support uniform epithelial maturation and stable monolayer formation, demonstrating their potential as engineered bioactive substrates [10]. Owing to their dynamic responsiveness to chemical and physical stimuli, such interfaces can also serve as reservoirs or delivery media for gases and hydrophobic molecules [11]. Incorporating a liquid–liquid interfacial strategy into microfluidic cell culture systems may therefore offer an alternative to porous membrane-based designs and mitigate the inherent limitations of synthetic solid membranes in gut-on-a-chip platforms. Among candidate materials, perfluorocarbon liquids such as FC-43 are particularly attractive due to their chemical inertness, immiscibility with aqueous media, and high gas solubility [11], making them well suited for multiphase microfluidic applications.

**Figure 1:**
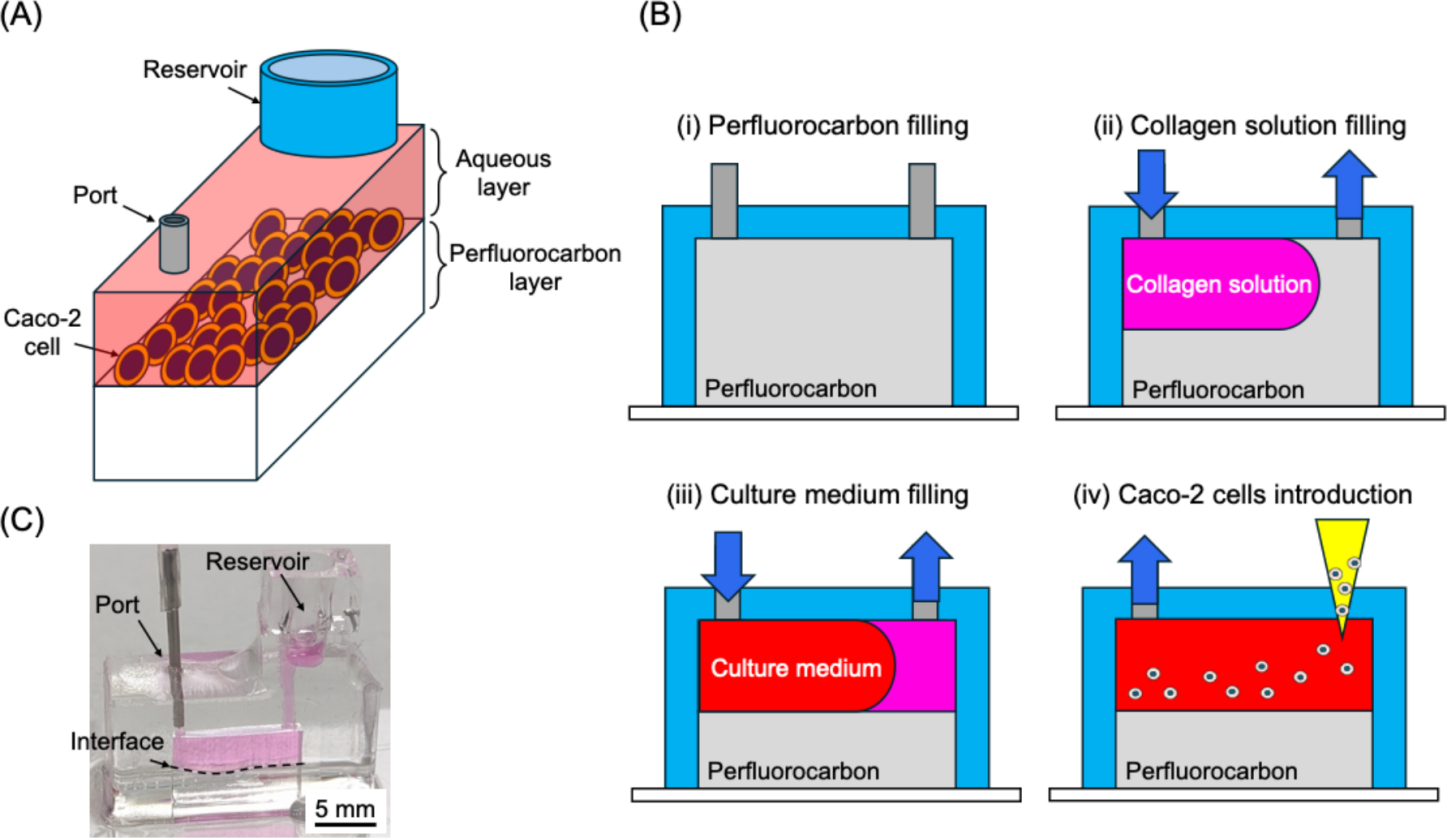
Illustration of the liquid–liquid interface formation. (A) Concept of the microfluidic device. (B) Fluid operation description: (i) FC-43 filling, (ii) collagen solution filling, (iii) culture medium filling, and (iv) Caco-2 cells introduction. (C) Fabricated microfluidic device. The dashed line indicates the boundary of the liquid–liquid interface.

In this study, we propose a novel microfluidic cell culture system by utilizing a collagen-coated liquid–liquid interface that serves as a cell-adhesion substrate for epithelial cells (Figure 1A). The rectangular cuboid-shaped microfluidic device allows for stable formation of the liquid–liquid interface for cell culture by sequential flow manipulation (Figure 1B). Using Caco-2 cells, a well-known epithelial cell line, we demonstrate that cells can adhere and form monolayers on the liquid–liquid interface. Immunofluorescence images reveal the formation of tight junctions and organized actin cytoskeletons, indicating epithelial early-stage maturation. Our results establish liquid–liquid interfaces as viable and functional substrates for epithelial cell culture and highlight their potential for the development of flexible, multiphase microfluidic models of epithelial barrier.

## Results and Discussion

### Liquid–liquid interface formation in the microfluidic device

The liquid–liquid interface was formed by first filling the microchannel with the perfluorocarbon liquid FC-43, followed by the introduction of an aqueous solution containing collagen (Figure 1B-ii). The effect of channel dimensions on the formation of the liquid–liquid interface was evaluated using the rectangular cuboid-shaped microfluidic device.

**Figure 2:**
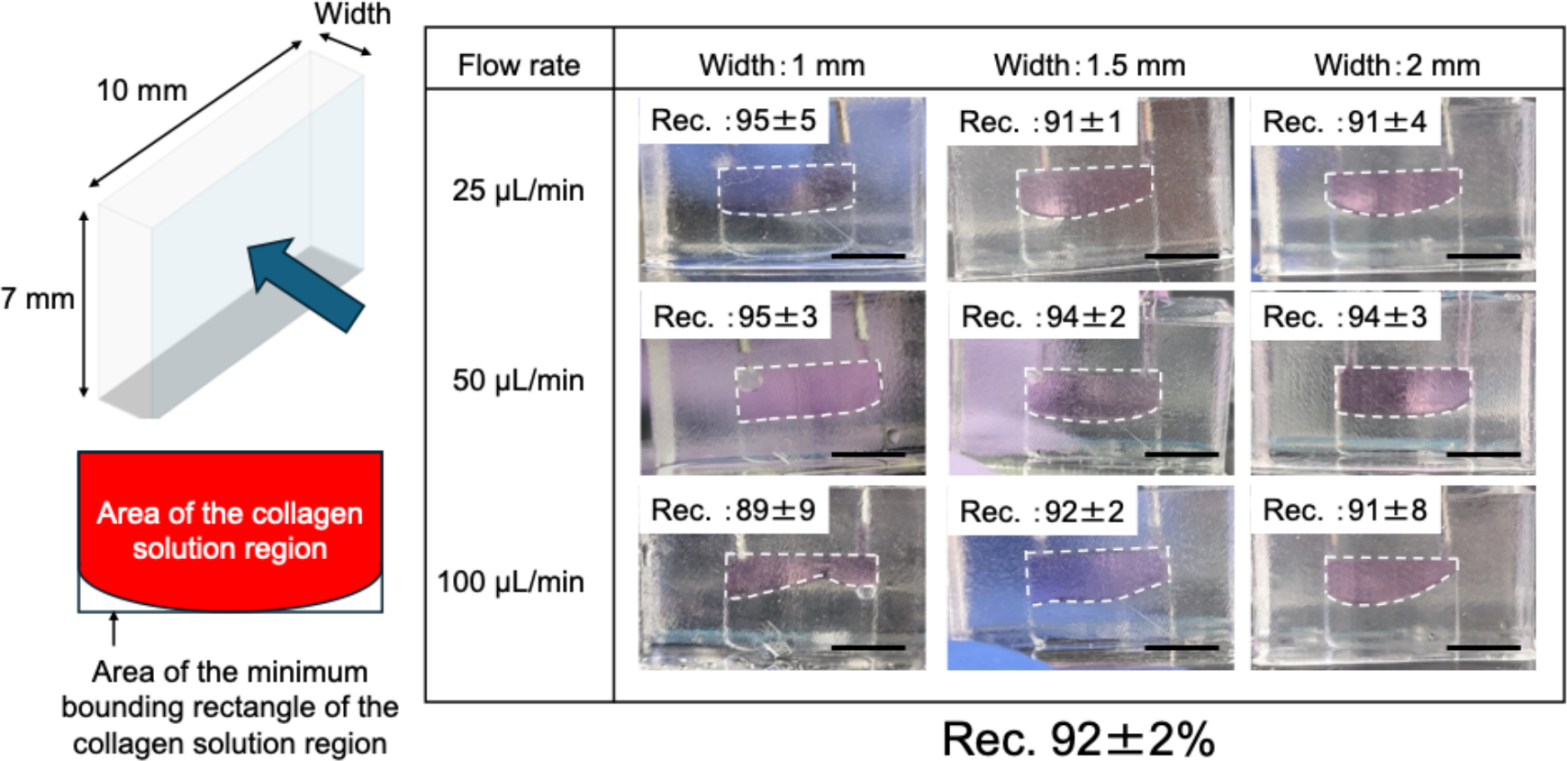
Rectangularity of the collagen solution region with respect to channel dimension (7 mm × 10 mm × width) and collagen solution introduction flow rate. The collagen region was imaged by a smartphone followed by the direction indicated by arrow. Rectangularity is abbreviated as “Rec”.

**Figure 3:**
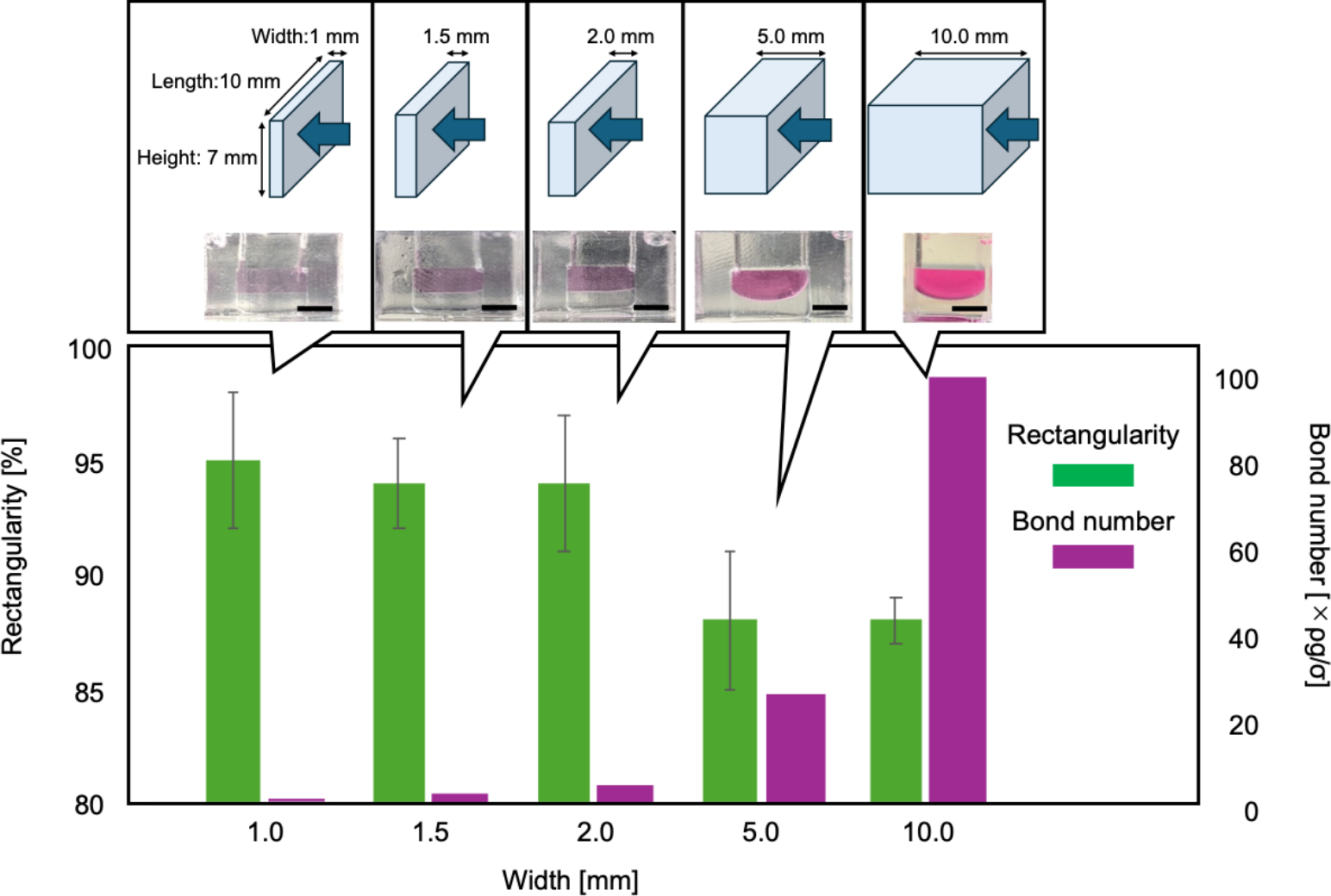
Rectangularity and Bond number as functions of the channel width (1.0, 1.5, 2.0, 5.0, and 10.0 mm). Representative interface images are shown above each bar. The images of the collagen solution region were captured from the direction indicated by the arrows. Scale bars are 5 mm. *n* = 3.

First, channel height and length were fixed at 7 and 10 mm, respectively, while the channel width was varied (1.0, 1.5, 2.0, 5.0, and 10.0 mm). Liquid–liquid interfaces were subsequently formed in each channel by introducing collagen solution at 50 µL/min. Here, when forming the liquid–liquid interface, a meniscus develops. To quantify the extent of this meniscus, we introduced rectangularity as an easily interpretable indicator. Rectangularity is calculated using Equation 1 based on the collagen region observed on the channel height × channel length plane of the microfluidic device (i.e., 7 mm × 10 mm in this case, see Figure 2, Figure 3, and Supporting Information File 1, Figures S1–S3) and the minimum bounding rectangle of the collagen solution region.


[1] $$\text{Rectangularity }(\%)=\frac{\text{The area of the collagen solution region}}{\text{The area of the minimum bounding rectangle of the collagen solution region}}\times 100$$

The rectangularity for each channel size is shown in Figure 3. When the channel width ranged from 1 to 2 mm, the rectangularity of the aqueous layer was 94% or higher; however, when the channel width was 5 or 10 mm, the rectangularity decreased to 88% (Figure 3). These results suggest that channel width affects the formation of the liquid–liquid interface. To analyze this effect, the “Bond number” (*B*_o_), a dimensionless parameter defined as


[2] $$B_{0}=\frac{\rho gL^{2}}{\sigma },$$

was introduced to evaluate whether gravitational forces or interfacial tension dominate when two fluids of different densities are in contact. Here, ρ denotes the density difference between FC-43 and the collagen solution, *g* is the gravitational acceleration, *L* is the channel width, and σ is the interfacial tension coefficient. In this experiment, as the channel width *L* was varied, its effect on the Bond number was directly assessed. Taking the Bond number for a channel width of 1 mm as a reference, the Bond numbers for channel widths of 1.5, 2, 5, and 10 mm were 2.25, 4, 25, and 100 times greater, respectively. For channel widths of 5 and 10 mm, the Bond number increased by one to two orders of magnitude. As the Bond number increases, gravitational forces increasingly dominate over interfacial tension. Thus, for channel widths of 5 and 10 mm, the gravitational effect on the aqueous layer exceeds that of surface tension, resulting in a pronounced meniscus and a reduction in rectangularity. In this study, a high rectangularity was interpreted as indicating an interface nearly parallel to the channel bottom, which is favorable for microscopic observation; accordingly, devices exhibiting high rectangularity were selected for the demonstration. For a practical application, the device with a width of 2 mm was selected for the cell culture experiments because it offered advantages in operation, including easier bubble removal.

**Figure 4:**
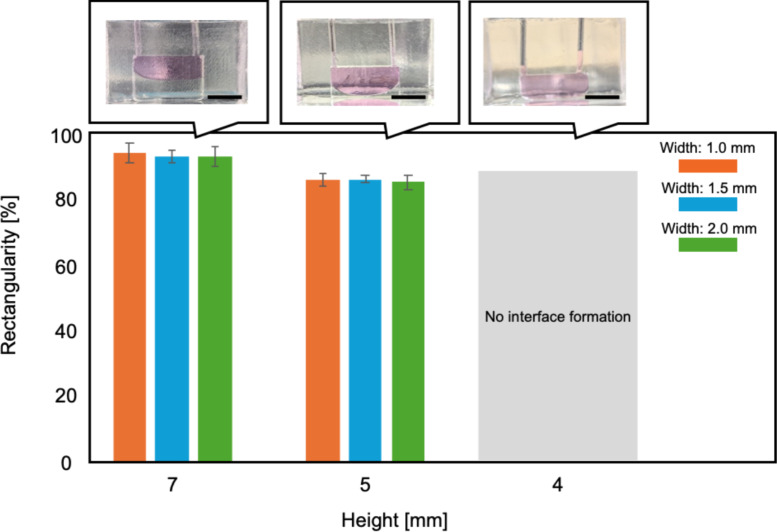
Rectangularity as a function of channel height (7, 5, and 4 mm). Representative interface images are shown above each bar. For a channel height of 4 mm, almost all of the FC-43 was replaced by the collagen solution; therefore, the rectangularity was not calculated, and it is labeled as “No interface formation”. Scale bars are 5 mm. *n* = 3.

In addition, the effect of channel height on the formation of the liquid–liquid interface was evaluated (Figure 4). The channel height was varied at 7, 5, and 4 mm. When the height was 4 mm, the FC-43 layer was almost completely displaced upon the introduction of the collagen solution, preventing interface formation. At a height of 5 mm, the liquid–liquid interface was formed, but the rectangularity decreased to approximately 87%, comparable to the rectangularity observed for aqueous layers in channels 5 and 10 mm wide at a height of 7 mm. These results indicate that channel height is also a critical parameter for the formation of the liquid–liquid interface.

Rectangularity was evaluated by varying both the channel size and the flow rate of the collagen solution (Figure 2, Supporting Information File 1, Figures S1–S3). Our results suggest that interface formation is largely insensitive to the flow rate of the collagen solution, whereas the channel size significantly governs the interfacial shape.

### Caco-2 cell culture on the liquid–liquid interface

**Figure 5:**
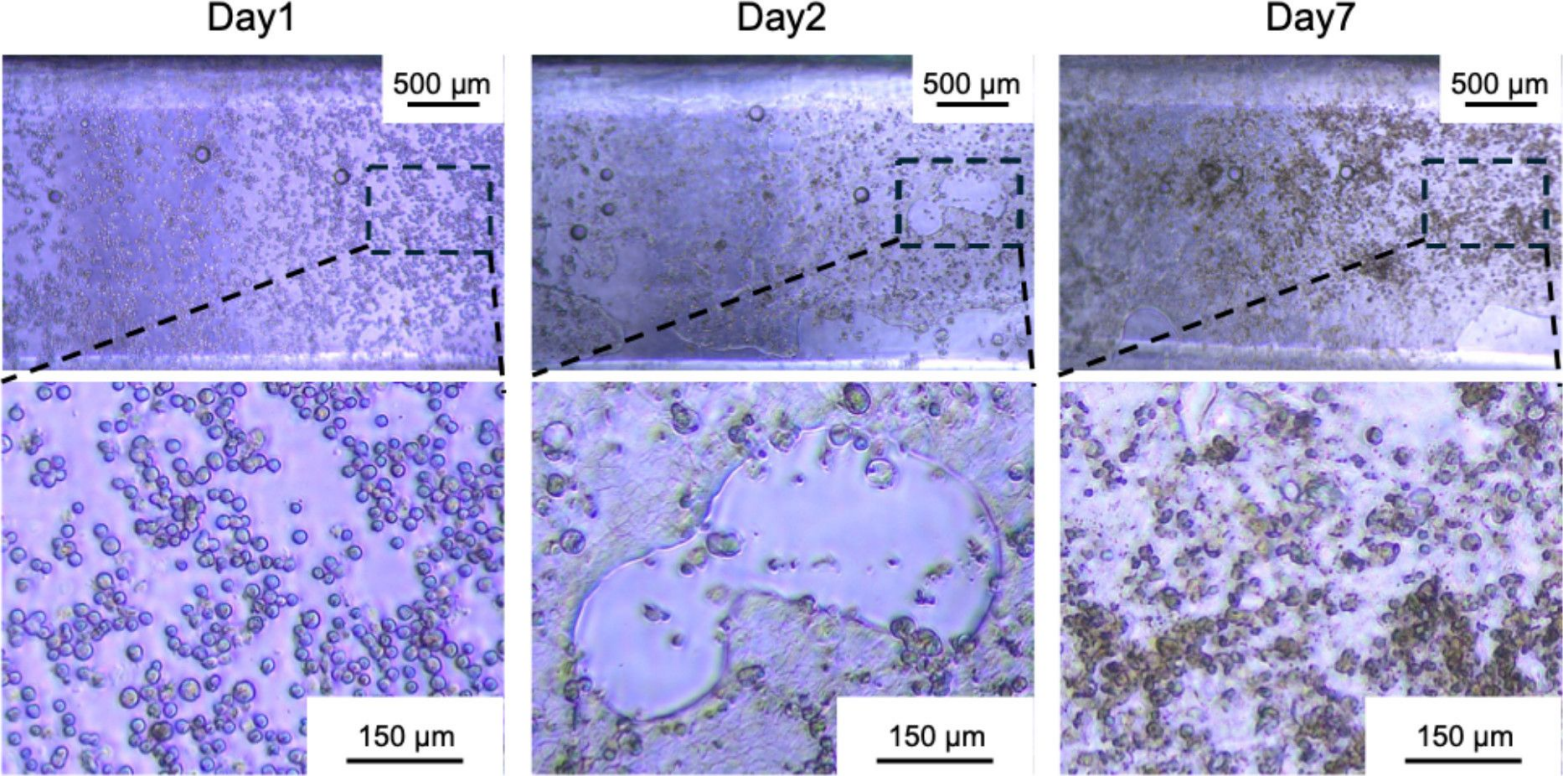
Time-lapse images of the progression of Caco-2 cells at the liquid–liquid interface. Images indicate Day1, Day2 and Day7, respectively. Region with a lower number of cells is magnified to show that Caco-2 cells grow on the interface.

The feasibility of adhesive cell culture on the liquid–liquid interface with a collagen layer was demonstrated by culturing Caco-2 cells (a human colorectal adenocarcinoma cell line). After forming the liquid–liquid interface using FC-43 and collagen-containing serum-free DMEM, the cell suspension was introduced and seeded at a density of 1.5 × 10^5^ cells/cm^2^. The growth of Caco-2 cells on the liquid–liquid interface is shown in Figure 5. The images were captured near the center of the channel. At two days post-seeding, most Caco-2 cells had adhered to the interface, resulting in the formation of a monolayer. This monolayer was maintained for seven days. In this study, Caco-2 cell monolayer culture was performed using three independent devices. As a result, confluency reached 95 ± 3% after seven days of culture. Here, the images of the left, center and right regions of the channel for each device were acquired on day seven of culture (see Supporting Information File 1, Figure S4), and confluency was calculated. Previous studies have suggested that Caco-2 cells begin to exhibit early functional characteristics of the intestinal epithelium, such as tight junction formation, after approximately seven days of culture [12]. In the present study, we therefore chose a period of seven days of culture to assess the early-stage maturation of Caco-2 monolayers from a proof-of-concept perspective. Here, the liquid–liquid interface remained intact and was stably maintained throughout the cell culture period (data is not shown).

To observe whether collagen was coated on the liquid–liquid interface, immunostaining for collagen was performed (Supporting Information File 1, Figure S5). Compared to the device without collagen coating, fluorescence was observed in the collagen-coated device. In addition, to evaluate the effect of collagen coating on the liquid–liquid interface, the behavior of Caco-2 cells seeded onto a liquid–liquid interface without collagen coating is shown in Supporting Information File 1, Figure S6. Caco-2 cells did not adhere on the liquid–liquid interface to form a monolayer; instead, they aggregated and were observed floating on the liquid–liquid interface. Those results indicate that bringing the collagen solution into contact with FC-43 results in the formation of a collagen layer on the liquid–liquid interface that is sufficiently robust to support cell adhesion.

### Caco-2 cells monolayer maturation

**Figure 6:**
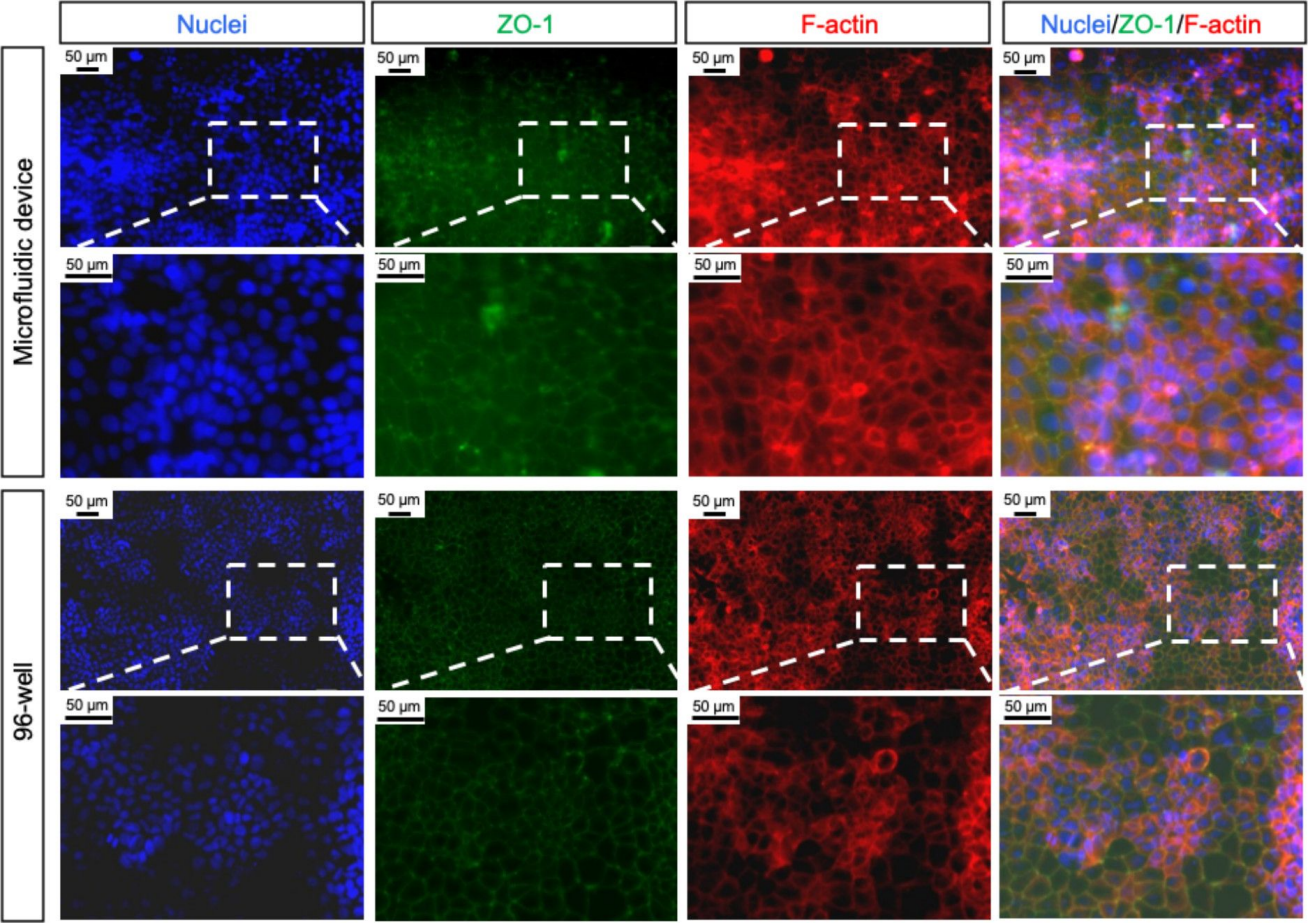
Immunofluorescence images of Caco-2 cells cultured in a microfluidic device and 96-well plates. Nuclei, ZO-1, F-actin and Merge images are displayed.

Immunofluorescence staining was performed to observe the characteristics of cultured Caco-2 cells as an intestinal epithelial model. Figure 6 shows a comparison of immunofluorescence images of ZO-1, F-actin, and nuclei in Caco-2 cells cultured in the microfluidic device and in 96-well plates. Under both conditions, epifluorescence imaging shows that the nuclei, ZO-1, and F-actin signals are localized within the same focal plane and are distributed across the entire liquid–liquid interface formed in the microfluidic device, indicating the formation of a monolayer. Although autofluorescence from FC-43 was observed in the images of the microfluidic devices, ZO-1 localization at cell–cell junctions was confirmed, suggesting the formation of epithelial barrier properties in Caco-2 cells cultured in the microfluidic device [13–14]. F-actin localization was observed under both conditions, suggesting that the cytoskeletal organization supporting the epithelial structure was maintained in Caco-2 cells cultured in the microfluidic device. Colocalization of ZO-1 with F-actin at cell–cell borders indicates the formation of mature tight junctions and the establishment of a functional epithelial barrier. Taken together, these results suggest that Caco-2 cells [15–16] cultured in the microfluidic device formed an epithelial monolayer with tight junctions and supporting cytoskeletal organization, recapitulating intestinal epithelial characteristics. Caco-2 cell culture and immunostaining within the microfluidic device were conducted in triplicate (*n* = 3), and representative results are shown in this study.

## Conclusion

We propose a unique rectangular cuboid-shaped microfluidic device that enables Caco-2 cell culture on a liquid–liquid interface. The collagen-coated liquid–liquid interface was formed by sequentially perfusing collagen-containing aqueous solution into the microfluidic channel filled with perfluorocarbon FC-43. Caco-2 cells were successfully cultured on the interface by forming a monolayer, and immunofluorescence imaging confirmed tight-junction formation and organized actin cytoskeletons, indicating early-stage epithelial maturation. Unlike conventional systems that rely on plastic or silicone rubber substrates for cell adhesion, our system enables direct cell culture on a liquid–liquid interface, offering a novel platform with potential for advanced drug transport studies and co-culture experiments with other cell types. This approach is expected to open new avenues for functional assays, such as transepithelial electrical resistance or drug permeability studies and co-culture experiments with other cell types.

## Experimental

### Device materials, reagents, and Caco-2 cells

The materials for device fabrication and assembly were obtained from the following suppliers: PDMS (Silpot 184W/C, Dow Corning Toray, Tokyo, Japan) and cover glass (MICRO COVER GLASS, MATSUNAMI GLASS IND., LTD., Osaka, Japan). Reagents for experiments were obtained from the following suppliers: formaldehyde (FA) (Formaldehyde, FUJIFILM Wako Pure Chemical Corporation, Osaka, Japan), Triton X-100 (Triton^TM^X-100, Sigma-Aldrich, St. Louis, MO, USA), ZO-1 antibody (ZO-1 Monoclonal Antibody [ZO1-1A12], Alexa Fluor^TM^ 488, Thermo Fisher Scientific Inc., Waltham, MA, USA), phalloidin (Phalloidin, Red Fluorescent Dye Conjugate, Acti-stain 555, Cytoskeleton, Inc., CO, USA), Hoechst33342 (Hoecst 33342 solution, Dojindo Laboratories, Kumamoto, Japan), PBS (D-PBS(−) (10×), Nacalai Tesque, Inc., Kyoto, Japan), DMEM (DMEM(1×)+GlutaMAX^TM^-I, Gibco, Thermo Fisher Scientific Inc., Waltham, MA, USA), FBS (Fetal Bovine Serum, Gibco, Thermo Fisher Scientific Inc., Waltham, MA, USA), Collagen type I-C (Cellmatrix^®^ Type I-C, Nitta Gelatin Inc., Osaka, Japan) and FC-43 (3M, St. Paul, MN, USA).

### Device fabrication

The microfluidic device was fabricated by following soft lithography. The polyacetal plate was milled to fabricate the molds, which were designed by three-dimensional computer-aided design software. The fabrication process is shown in Supporting Information File 1, Figure S7. Briefly, PDMS was cast on molds and cured at 85 °C for 5 h (Figure S7i). The cured PDMS was removed from the mold and holes with diameters of 1.0 and 1.5 mm were punched for port and reservoir, respectively (Figure S7ii). Port and reservoir were attached on to the microfluidic device (Figure S7iii). A thin layer of uncured PDMS was applied to the bottom of the device, attached to a coverslip and cured at 85 °C for 2 h (Figure S7iv).

### Caco-2 cell culture

The procedure of the Caco-2 cell culture in the microfluidic device is shown in Figure S8. First, the device was filled with FC-43 (Figure S8i). Next, collagen-containing serum-free DMEM (60 μg/mL) was introduced into the channel at a flow rate of 50 μL/min using a syringe pump (KDS, kd Scientific, MA, USA) from the port (Figure S8ii). In this study, the collagen concentration was selected as the optimal condition based on preliminary experiments (data not shown). In brief, excessively low collagen concentrations impede cell adhesion, while excessively high concentrations lead to gelation, making fluid handling impossible. The microfluidic device was put in the refrigerator (4 °C, 2 h) (Figure S8iii). Thereafter, culture medium was introduced into the channel at a flow rate of 10 μL/min for 10 min to replace the existing liquid layer (Figure S8iv). The human large intestinal cancer cell line Caco-2 cells (CACO-2, KAC Co., Ltd., Kyoto, Japan) were harvested and resuspended in culture medium (DMEM/FBS = 9:1) (Figure S8v). A portion of the cell suspension was aspirated using a pipette, and the pipette tip was mounted onto the device reservoir. The cell suspension was then introduced into the channel from the port using a syringe pump at a flow rate of 20 μL/min for 1 min, and Caco-2 cells were seeded onto the liquid–liquid interface at a density of 1.5 × 10^5^ cells/cm^2^. The microfluidic device was then put in an incubator (37 °C, 5% CO_2_). The culture medium was replaced daily by flowing fresh medium through the channel at a flow rate of 10 μL/min for 10 min. For control experiments, Caco-2 cells were seeded into 96-well plates at a density of 1.5 × 10^5^ cells/cm^2^, and the culture medium was replaced daily. The microfluidic device and the 96-well plate were put in an incubator (37 °C, 5% CO_2_).

### Immunostaining procedure

For immunostaining, Caco-2 cells cultured in the channel device and 96-well plates for seven days were fixed with 4% FA/PBS for 20 min, followed by permeabilization with 0.5% Triton X-100/PBS at room temperature for 20 min (Supporting Information File 1, Figure S9ii,iv). Blocking was performed with 1% BSA/PBS at 4 °C overnight (Figure S9vi). The samples were then incubated at room temperature for 3 h in a solution containing ZO-1 antibody (5 μg/mL), phalloidin (0.1 μM), and Hoechst33342 (2 μg/mL). Between each procedure, PBS was filled to wash the channel or wells (Figure S9i,iii,v,vii).

For immunostaining, the collagen layer coated on the liquid–liquid interface was fixed with 4% FA/PBS for 20 min at room temperature for 20 min (Figure S10v). Blocking was performed with 1% BSA/PBS at 4 °C overnight (Figure S10vii). The samples were then incubated at room temperature for 3 h in a solution containing collagen antibody (5 μg/mL) (Figure S10ix). In the negative control experiment without collagen coating, the procedure was carried out in the same manner, except for the step shown in Figure S10ii.

### Image acquisition and quantification

The appearance of the aqueous layer was captured using a smartphone (Google Pixel 9a, Google LLC, CA, USA). Bright-field images of the cells were acquired using an inverted microscope (Leica DMil, Leica Microsystems, Wetzlar, Germany) equipped with a FLEXACAM camera. Fluorescence images were acquired using an all-in-one fluorescence microscope (BZ-X700, Keyence Corporation, Osaka, Japan). The fluorescence images were saved in 14-bit TiFF format, and the images were processed using the ImageJ software (NIH).

## Supporting Information

File 1 Additional figures.

## Data Availability

All data that supports the findings of this study is available in the published article and/or the supporting information of this article.
