# Supplementary material for: Interface-engineered Caco-2 cell culture on a collagen-coated liquid–liquid interface in a microfluidic device
Source: Beilstein J Nanotechnol. 2026 Jun 11;17:760–8. doi: 10.3762/bjnano.17.53 (PMC13267505; doi:10.3762/bjnano.17.53)
Supplement: File 1 — Additional figures. [file Beilstein_J_Nanotechnol-17-760-s001.pdf]

BEILSTEIN JOURNAL OF NANOTECHNOLOGY

Supporting Information

for

Interface-engineered Caco-2 cell culture on a collagen-coated liquid–liquid interface in a microfluidic device

Satoru Kuriu and Soo Hyeon Kim

Beilstein J. Nanotechnol. **2026,** *17,* 760–768. doi:10.3762/bjnano.17.53

Additional figures

License and Terms: This is a supporting information file under the terms of the Creative Commons Attribution License (https://creativecommons.org/licenses/by/4.0). Please note that the reuse, redistribution and reproduction in particular requires that the author(s) and source are credited and that individual graphics may be subject to special legal provisions.
The license is subject to the *Beilstein Journal of Nanotechnology* terms and conditions: (https://www.beilstein-journals.org/bjnano/terms)

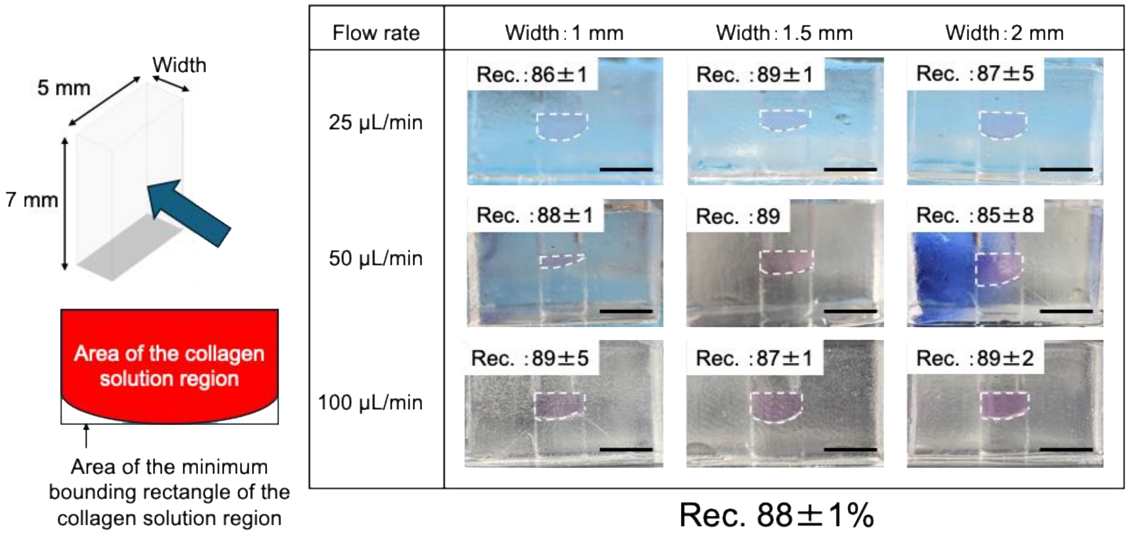

Figure S1: Rectangularity of the collagen solution region with respect to channel dimension (7 × 5 × width) and collagen solution introduction flow rate. Collagen region was imaged by smartphone followed by the direction indicated by arrow. Rectangularity is abbreviated as “Rec”.

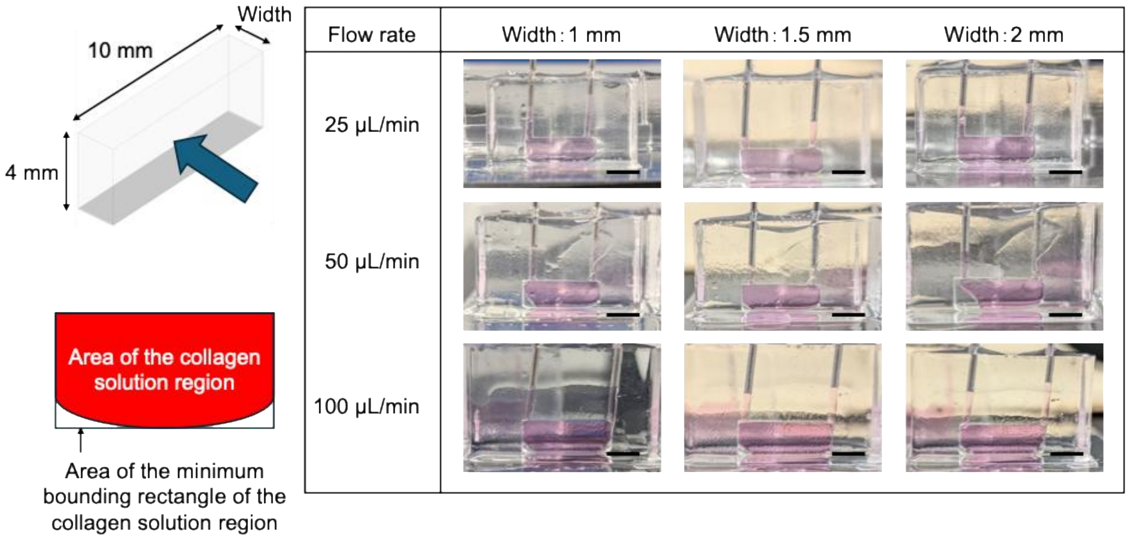

Figure S2: The collagen solution region with respect to channel dimension (4 × 10 × width) and collagen solution introduction flow rate. Collagen region was imaged by smartphone followed by the direction indicated by arrow. For a channel height of 4 mm, almost all of the FC-43 was replaced by the collagen solution.

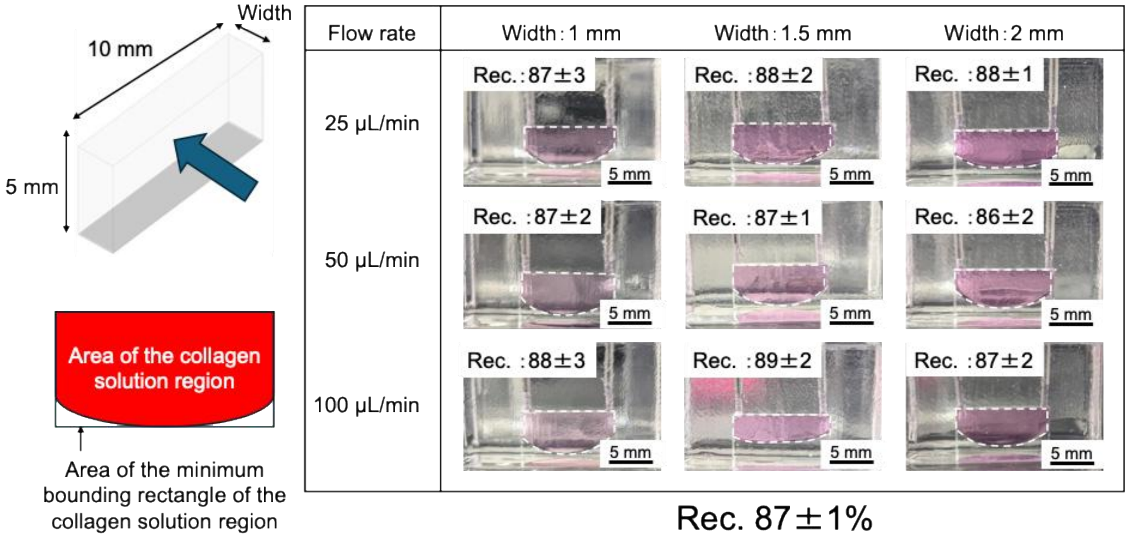

Figure S3: Rectangularity of the collagen solution region with respect to channel dimension (5 × 10 × width) and collagen solution introduction flow rate. Collagen region was imaged by smartphone followed by the direction indicated by arrow. Rectangularity is abbreviated as "Rec".

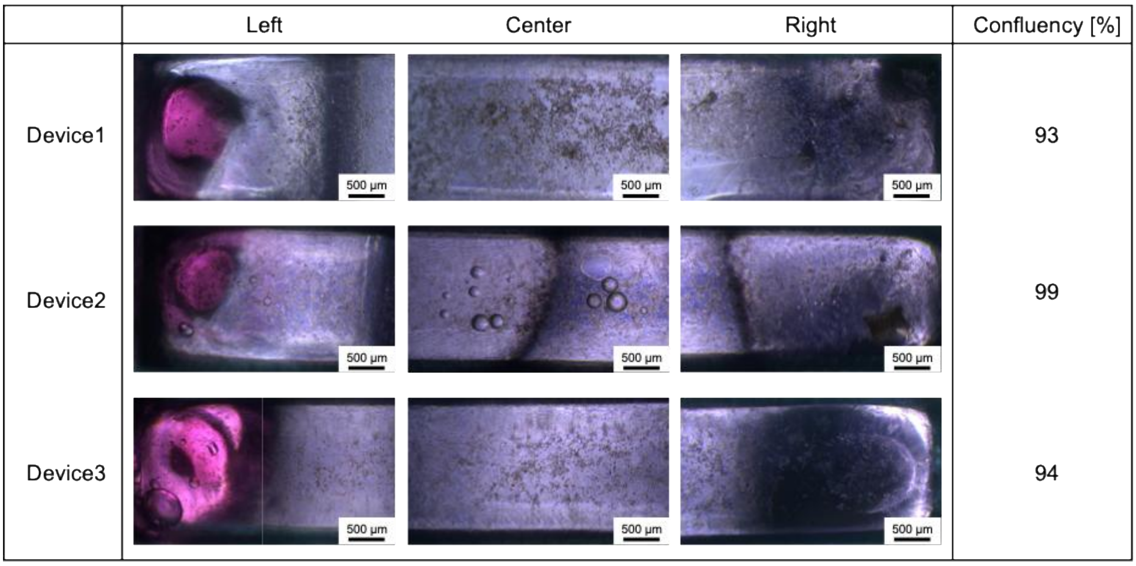

Figure S4: Caco-2 cell monolayers formed on the liquid-liquid interface. Images of the left, center and right regions of the channel were acquired on day 7 of culture. The confluency for each independent device is indicated.

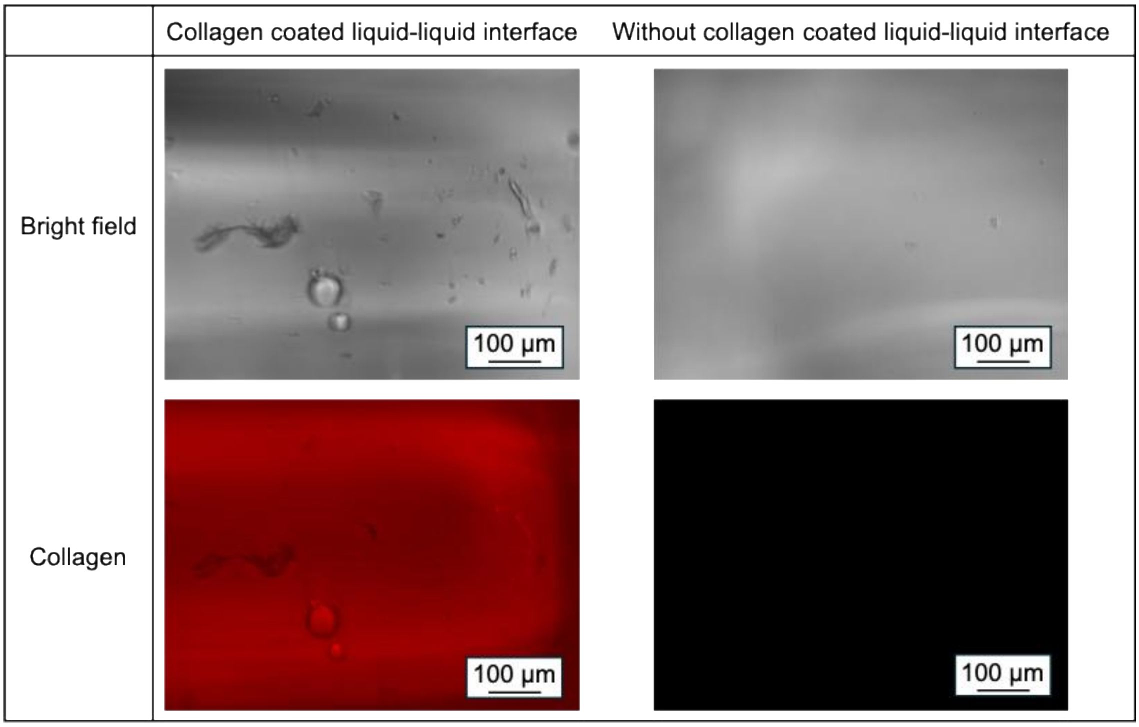

Figure S5: Immunofluorescence images of liquid-liquid interface with collagen layer and without collagen layer. The fluorescently labeled anti-collagen antibody emitted red fluorescence by binding to the collagen layer coated on the liquid-liquid interface.

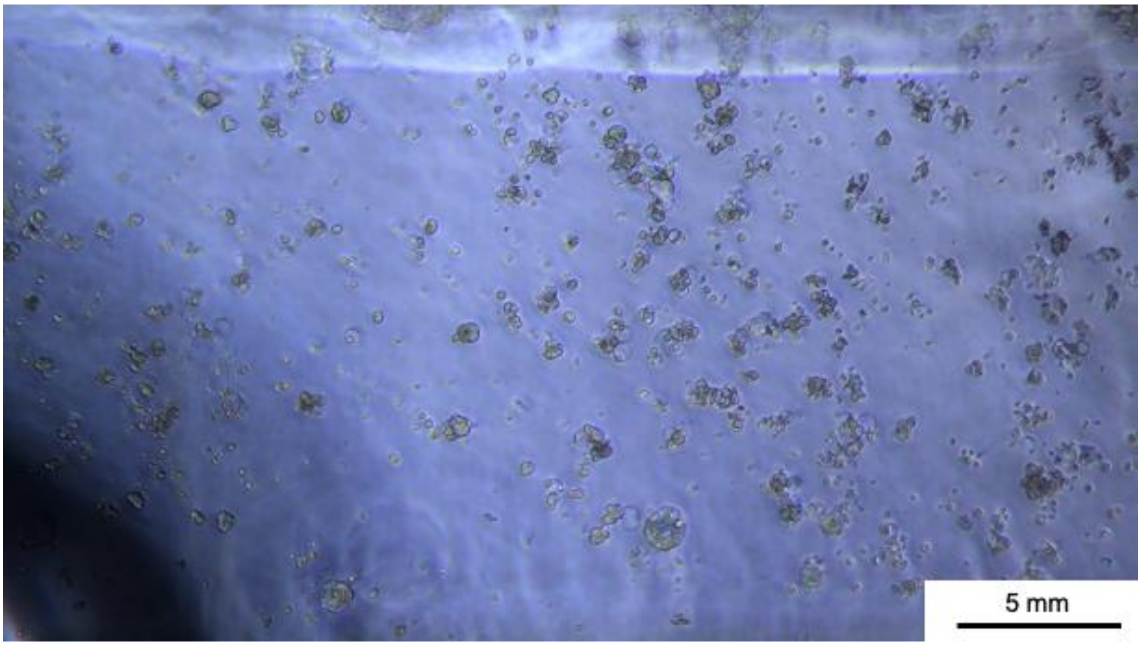

Figure S6: Caco-2 cells seeded onto a liquid-liquid interface without collagen coating. The image was captured 2 days post-seeding.

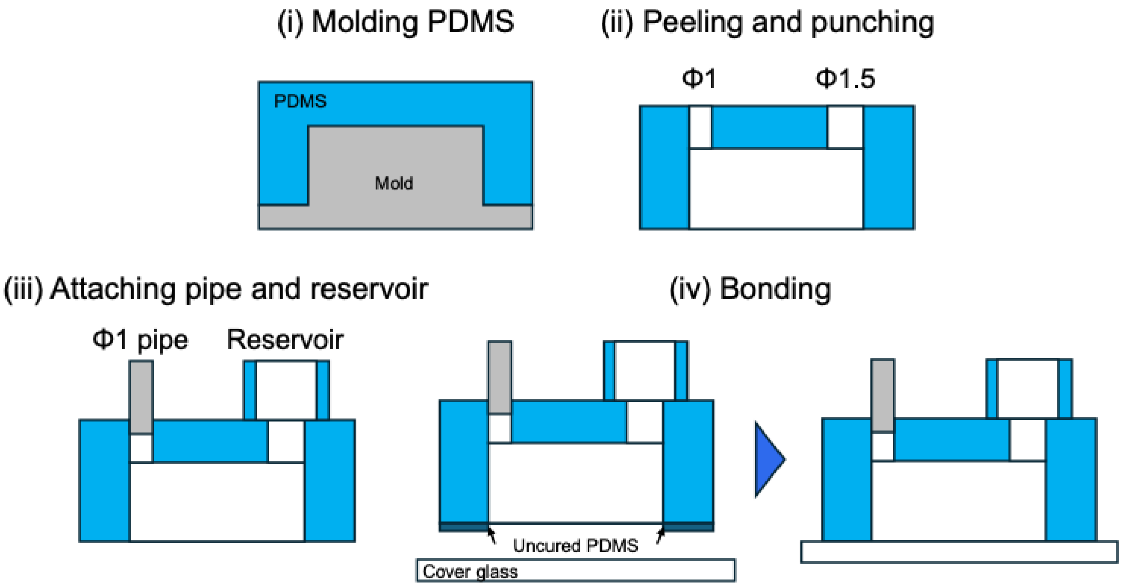

Figure S7: Fabrication procedure of the microfluidic device. (i) Molding PDMS. (ii) Peeling and punching. (iii) Attaching pipe and reservoir. (iv) Bonding.

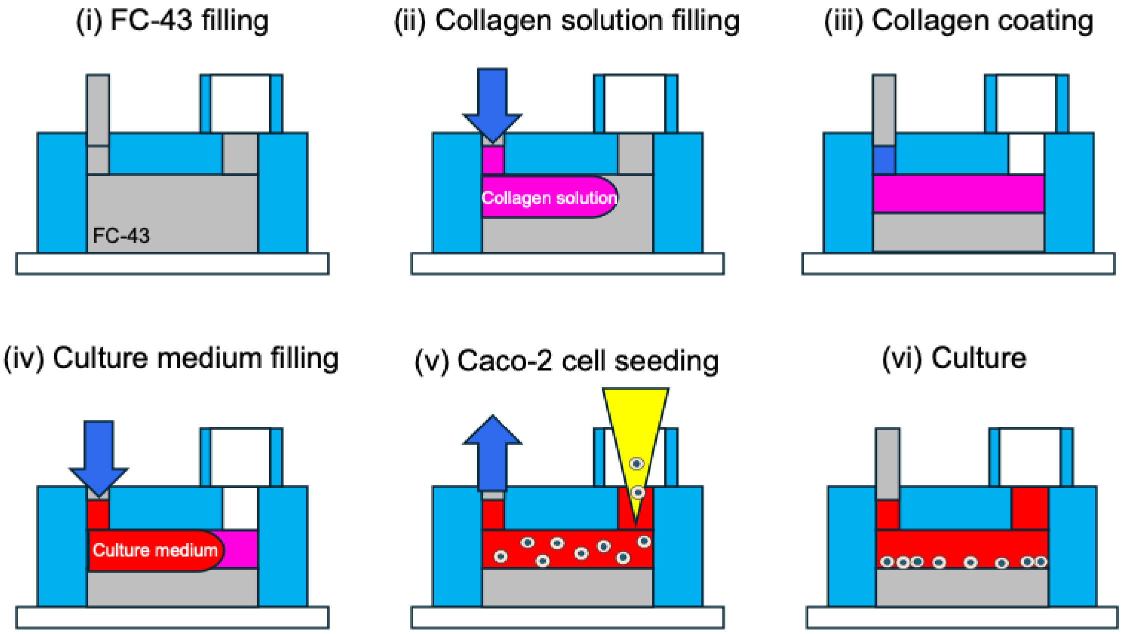

Figure S8: Caco-2 cell culture procedure in the microfluidic device. (i) FC-43 filling. (ii) Collagen solution filling. (iii) Collagen coating. (iv) Culture medium filling. (v) Caco-2 cell seeding. (vi) Culture.

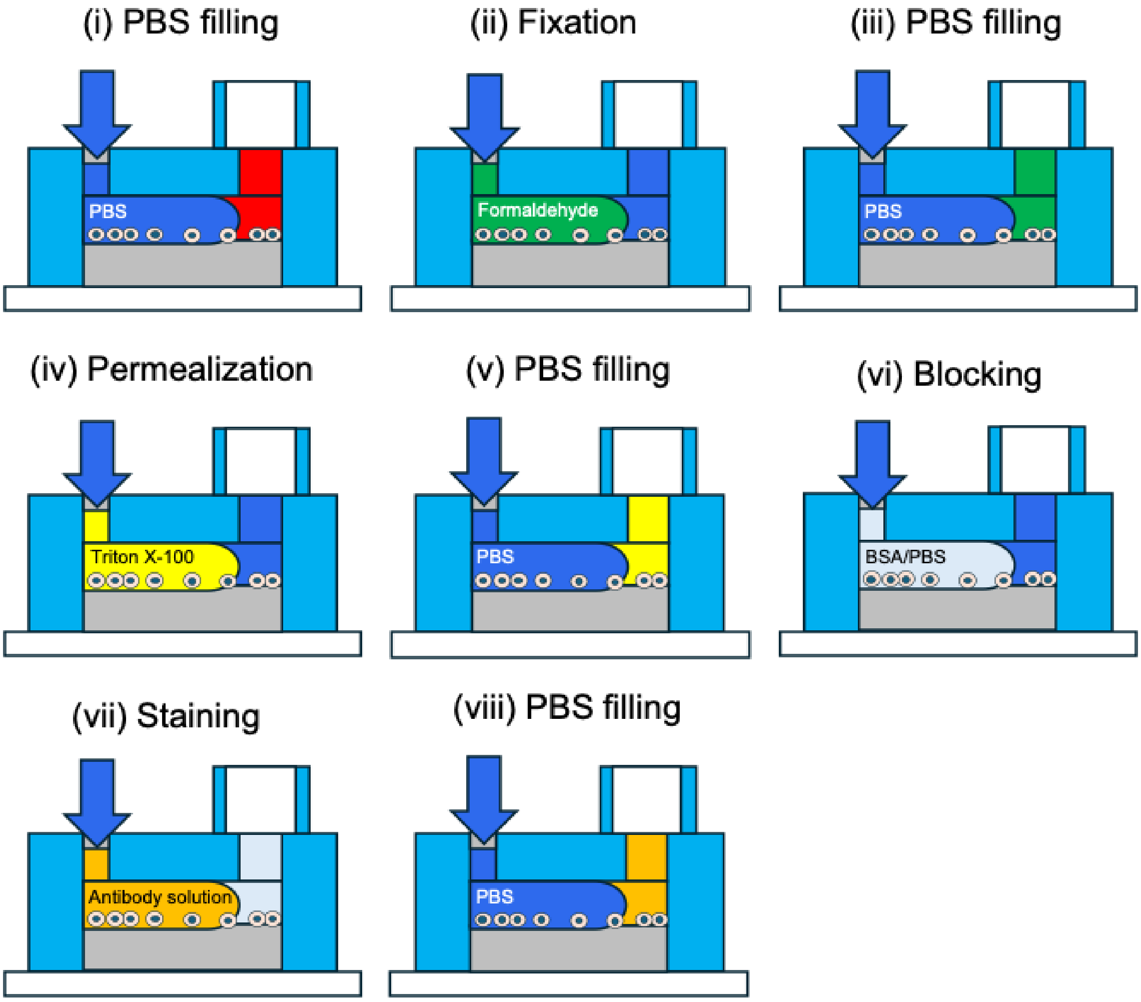

Figure S9: Immunostaining procedure in the microfluidic device. (i) PBS filling. (ii) Fixation. (iii) PBS filling. (iv) Permealization. (v) PBS filling. (vi) Blocking. (vii) Staining. (viii) PBS filling.

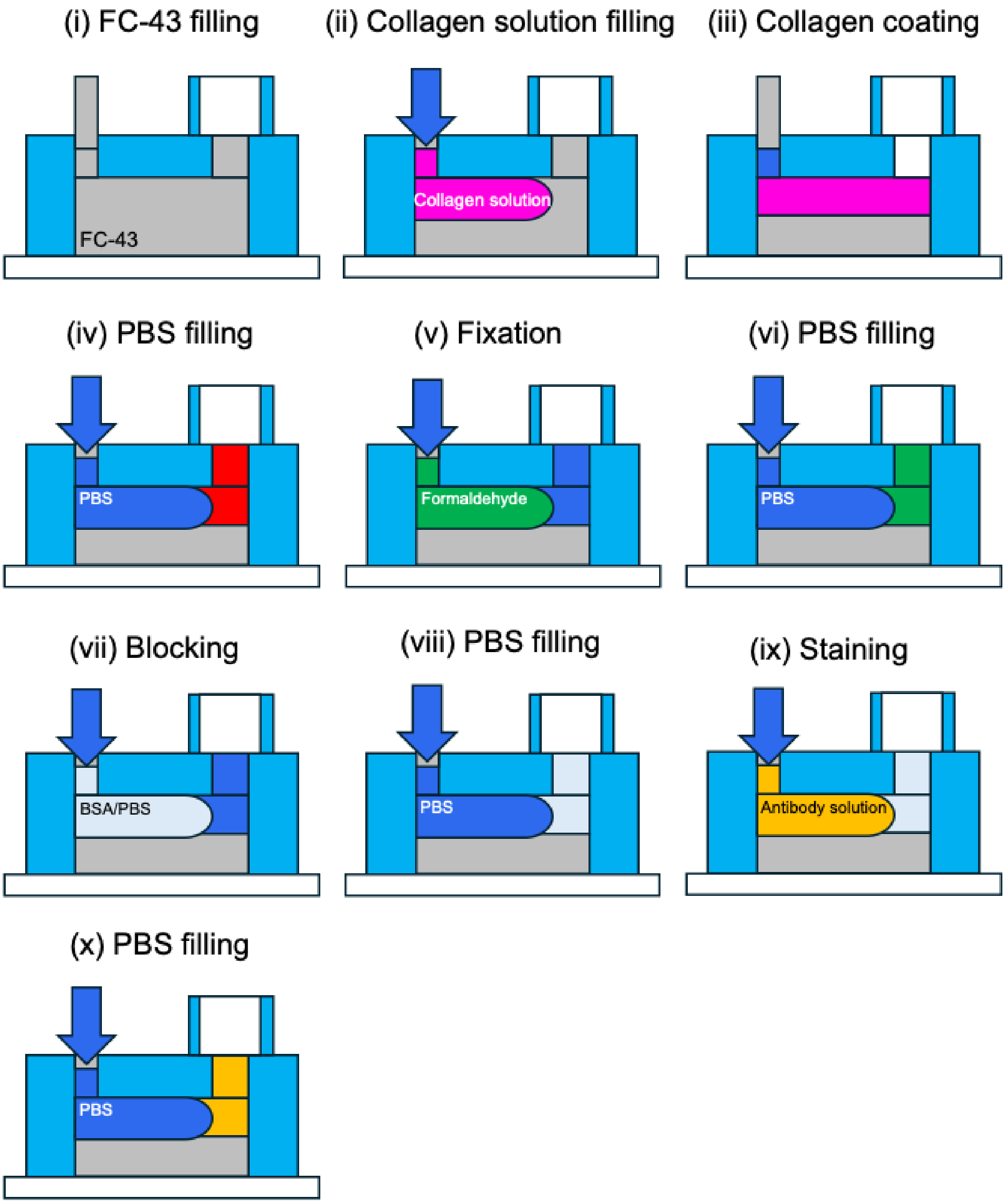

Figure S10: Immunostaining procedure for the collagen layer coated on the liquid-liquid interface. (i) FC-43 filling. (ii) Collagen solution filling. (iii) Collagen coating. (iv) PBS filling. (v) Fixation. (vi) PBS filling. (vii) Blocking. (viii) PBS filling. (ix) Staining. (x) PBS filling.
